# RNA Sequencing Identifies Upregulated Kyphoscoliosis Peptidase and Phosphatidic Acid Signaling Pathways in Muscle Hypertrophy Generated by Transgenic Expression of Myostatin Propeptide

**DOI:** 10.3390/ijms16047976

**Published:** 2015-04-09

**Authors:** Yuanxin Miao, Jinzeng Yang, Zhong Xu, Lu Jing, Shuhong Zhao, Xinyun Li

**Affiliations:** 1 Key Laboratory of Agricultural Animal Genetics, Breeding and Reproduction of Ministry of Education, Huazhong Agricultural University, Wuhan 430070, China; E-Mails: miaoyuanxin19880610@126.com (Y.M.); xz8907@163.com (Z.X.); deer66@126.com (L.J.); shzhao@mail.hzau.edu.cn (S.Z.); 2 The Cooperative Innovation Center for Sustainable Pig Production, Wuhan 430070, China; 3 Department of Human Nutrition, Food and Animal Sciences, University of Hawaii at Manoa, Honolulu, HI 96822, USA; E-Mail: jinzeng@hawaii.edu

**Keywords:** myostatin, RNA-sequencing, transgenic mice, muscle hypertrophy

## Abstract

Myostatin (MSTN), a member of the transforming growth factor-β superfamily, plays a crucial negative role in muscle growth. MSTN mutations or inhibitions can dramatically increase muscle mass in most mammal species. Previously, we generated a transgenic mouse model of muscle hypertrophy via the transgenic expression of the MSTN *N*-terminal propeptide cDNA under the control of the skeletal muscle-specific MLC1 promoter. Here, we compare the mRNA profiles between transgenic mice and wild-type littermate controls with a high-throughput RNA sequencing method. The results show that 132 genes were significantly differentially expressed between transgenic mice and wild-type control mice; 97 of these genes were up-regulated, and 35 genes were down-regulated in the skeletal muscle. Several genes that had not been reported to be involved in muscle hypertrophy were identified, including up-regulated myosin binding protein H (mybph), and zinc metallopeptidase STE24 (Zmpste24). In addition, kyphoscoliosis peptidase (Ky), which plays a vital role in muscle growth, was also up-regulated in the transgenic mice. Interestingly, a pathway analysis based on grouping the differentially expressed genes uncovered that cardiomyopathy-related pathways and phosphatidic acid (PA) pathways (Dgki, Dgkz, Plcd4) were up-regulated. Increased PA signaling may increase mTOR signaling, resulting in skeletal muscle growth. The findings of the RNA sequencing analysis help to understand the molecular mechanisms of muscle hypertrophy caused by MSTN inhibition.

## 1. Introduction

Myostatin, a member of the transforming growth factor-β superfamily and also known as growth factor and differentiation factor-8 (GDF-8), is an essential muscle endogenous negative regulatory factor secreted by skeletal muscles. Myostatin is mainly expressed in skeletal muscle tissue and negatively regulates muscle growth and development [1,2]. In 1997, MSTN was first confirmed as a muscle growth inhibitor in a knockout mouse model. As reported, the muscles of the myostatin-null mice weighed almost two times as much as those of the controls due to increased muscle fiber number and diameter [3,4,5]. Myostatin was highly conserved, and it can inhibit muscle growth in other mammalian species. The mutation and inhibition of MSTN can dramatically increase muscle mass in most mammal species. A natural mutation of myostatin in double-muscled cattle may account for the double muscling phenotype [6,7]. A genetic study confirmed that one G/A mutation identified in the 3' UTR of the myostatin gene formed a novel binding site for miR-1 and miR-206 in Texel sheep. These miRNAs inhibited the protein expression of MSTN, causing the muscle hypertrophy phenomenon in this sheep breed [8]. Furthermore, a mutant MSTN gene causes muscle hypertrophy in humans [9].

MSTN is synthesized as an inactive precursor protein, which is the predominant form in the muscle extracellular matrix and in circulation [10]. The precursor protein undergoes dimerization and two proteolytic cleavage processes to generate a *C*-terminal dimer—an active biological molecule. The dimer remains in an inactive, latent complex with the *N*-terminal pro-peptide and circulates in the blood [5]. The *C*-terminal dimer (mature myostatin) can be activated when separated with the *N*-terminal, and mature myostatin can bind to the transmembrane ActRIIA/B receptor, which then recruits the type I receptor, ALK-4/5, and phosphorylates the type I receptor to activate its kinase domains [11,12,13]. After binding the receptors, Smad2 and Smad3 can be activated to form a heterodimer complex with Smad4. The Smad complex then activates the Erk1/2 MAPK pathway, which can prevent the proliferation of myoblasts via the p21/Rb signaling cascade pathway and can promote anti-apoptotic pathways by activating p53 in differentiated cells. Moreover, activated Smad3 can repress the level of the muscle regulatory factors (MRFs) [14]. During the interference of Smad3, MyoD sequesters in the cytoplasm and does not enter the nucleus to activate myogenesis [15]. The activated Smad complex can activate Smad4 and cause it to translocate to the nucleus to block the transactivation of MyoD and activate the expression of Smad7 [11,16,17]. Furthermore, phosphorylated SMAD3 can inhibit the PI3K/Akt/mTOR signaling pathway via the induction of E3-ligase atrogin-1 [18,19]. A previous study also found that MSTN can induce muscle protein degradation by activating the ubiquitin-proteasome to inhibit the PI3K/Akt/mTOR signaling pathway [20].

Blocking the myostatin signaling transduction pathway can induce a highly muscled phenotype [21]. The ability of several myostatin antagonists, such as follistatin, follistatin-related gene, soluble activin receptor IIB and suramin to block MSTN signaling transduction has been tested [22,23,24,25]. Furthermore, some microRNAs can affect the MSTN signaling pathway by targeting the 3' UTR of myostatin [26,27]. We previously generated transgenic mice via the muscle-specific expression of a cDNA sequence (5'-region 886 nucleotides) encoding the MSTN propeptide. The transgene effectively depressed myostatin function, and transgenic mice showed dramatic growth and increases in muscle mass due to muscle fiber hypertrophy [28,29,30]. To further elucidate the mechanisms of muscle hypertrophy caused by MSTN inhibition, we compared the gene expression profiles of the muscle tissue between MSTN propeptide transgenic mice and their littermate wild-type controls. Our results indicate that the Ky/mybph/actins pathway is a novel noncanonical signaling pathway, and phosphatidic acid inhibits mTOR signaling via Dgki, Dgkz and Plcd4 in the hypertrophic skeletal muscle of the MSTN propeptide transgenic mice.

## 2. Results

### 2.1. Skeletal Muscle Weights of the Transgenic Mice and Wild-Type Littermate Controls

**Figure 1:**
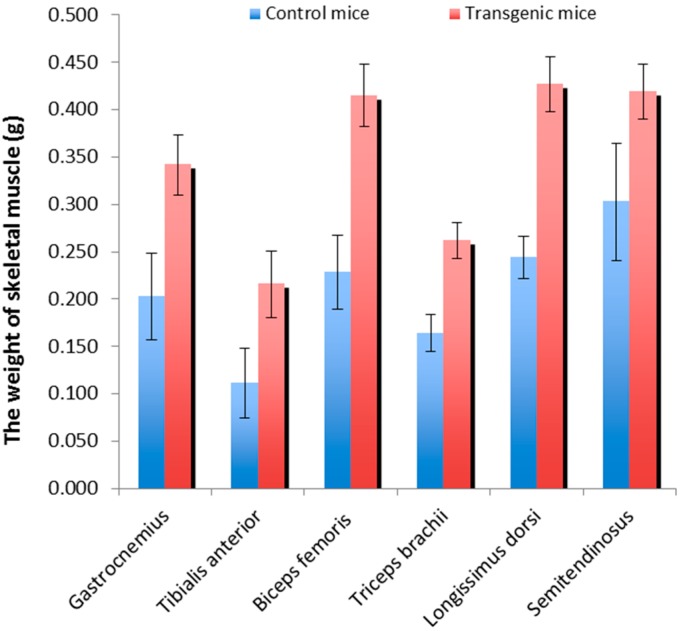
The skeletal muscle weights of the transgenic mice and wild-type littermate controls.

The mice were sacrificed at four months of age to evaluate the muscle weight and obtain muscle samples. The results were consistent with our previous report; the individual major muscles of the myostatin propeptide transgenic mice were significantly heavier (38% to 95% more muscle) than those of the wild-type littermate controls (Figure 1). The muscle groups that increased in mass predominantly consisted of fast-twitch muscle fibers, including the gastrocnemius, tibialis anterior, biceps femoris, triceps brachii, longissimus dorsi, and semitendinosus.

### 2.2. Solexa Sequencing Data and Reads Assembly Using Tophat Software

In this study, Solexa sequencing was used to detect the mRNA expression profiles of MSTN propeptide transgenic mice and their littermates. Five RNA libraries were constructed from the total RNA isolated from the gastrocnemius muscle of the transgenic and control mice, which generated 9,710,772 (CN148), 10,371,693 (CN164), 9,991,395 (TN126), 10,258,517 (TN135), and 10,076,991 (TN329) raw reads. After filtering the low quality reads and trimming off the adapted fragments, 8,717,967, 9,418,459, 9,054,439, 9,301,124, and 9,032,266 clean reads were obtained, and the ratios of cleans reads in raw reads were 89.78%, 90.81%, 90.62%, 90.67%, and 89.63% (Table 1). The detailed description of the sequence data is shown in Table 1, and the abundance of mRNA reads mapped to each chromosome is shown in Figure S1. The Tophat aligner software was used to align the sequencing reads to the reference genome download from the ENSEMBEL website ftp://ftp.ensembl.org/pub/release-75/fasta/mus_musculus/dna/. The mapping rate of each sample exceeded 97% (Table 2). The statistical alignment data indicated that the sequencing reads were of high quality and the sequencing depth was sufficient for a differential expression analysis between the two groups. Subsequently, HTSeq was selected to control the quality of aligned reads. The quality control result is shown in Figure S2.

**Table 1:** The overview of raw RNA sequencing data in muscle samples of propeptide transgenic and control mice.

	CN148	CN164	TN126	TN135	TN329
Raw reads	9,710,772	10,371,693	9,991,395	10,258,517	10,076,991
Clean reads	8,717,967	9,418,459	9,054,439	9,301,124	9,032,266
Ratio	89.78%	90.81%	90.62%	90.67%	89.63%

**Table 2:** The clean reads counts and alignment results of each library.

	CN148	CN164	TN126	TN135	TN329
Input	8,717,967	9,418,459	9,054,439	9,301,124	9,032,266
Output	8,717,523	9,418,247	9,054,198	9,300,876	9,031,776
Qualified	99.99%	99.99%	99.99%	99.99%	99.99%
Mapped	8,583,223	9,251,338	8,865,832	9,118,889	8,890,164
Mapped%	98.5%	98.2%	97.9%	98%	98.4%
Multiple alignments	2,249,697	1,869,472	1,801,533	1,789,715	2,407,543
Multiple alignments%	26.2%	20.2%	20.3%	19.6%	27.1%
Min read length	30	30	30	30	30
Max read length	50	50	50	50	50

### 2.3. Differentially Expressed Genes of Skeletal Muscle between Transgenic Mice and Their Littermate Controls

**Figure 2:**
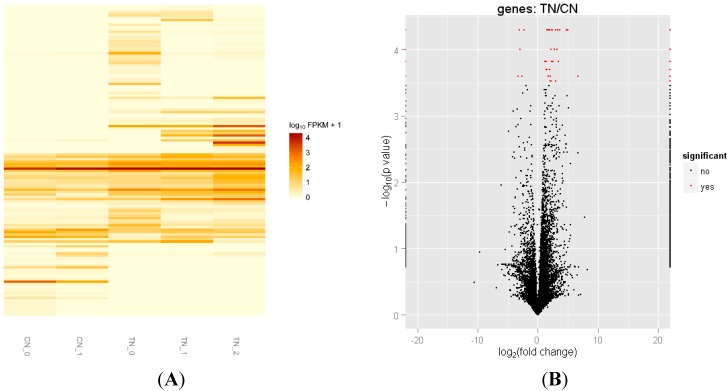
Differentially expressed genes between MSTN propeptide transgenic mice and their littermate control mice. (**A**) A heatmap of FPKM expression values in the five samples. Each row represents a gene ID and the column represents a sample; (**B**) The volcano plots reveal genes that differ significantly between two groups. The black dots represent the expression levels of genes that do not differ and the red dots represent the expression level differences.

The cufflinks software was used to assemble the individual transcripts that had been aligned to the genome and quantify the expression level of each transfrag in the sample. After cufflinks treated the reads, cuffmerge was used to parsimoniously merge the assembled transfrags. The annotation reference file of Mus musculus integrated into the merged assembly was downloaded from ENSEMBEL (ftp://ftp.ensembl.org/pub/release-75/gtf/mus_musculus/) [31]. Cuffdiff was used to calculate the differential expression and analyze the significances of observed changes between two groups. A cluster analysis was performed to elaborate the expression patterns of genes in MSTN propeptide transgenic mice and their controls (Figure 2A). The results showed that approximately 70% genes were up-regulated in the transgenic mice. CummeRbund volcano plots (Figure 2B) were available to intuitively observe the genes that were differentially expressed between the two groups. Finally, the expression levels of 132 genes were identified to be significantly different between the transgenic mice and control mice; among these genes, 97 were up-regulated and 35 were down-regulated (FDR < 0.05). Of the 132 genes, 117 were annotated in the database. The differentially expressed genes with more than two-fold changes and an FPKM value more than 5 are listed in Table 3. Several genes have not been reported to be involved in skeletal muscle hypertrophy, including myosin binding protein H (mybph), beta, gamma and alpha actin (Actb, actc1, actg1), protein tyrosine phosphatase receptor type c (Ptprc), zinc metallopeptidase STE24 (zmpste24), and genes related to phosphatidic acid (PA) pathways (Dgki, Dgkz and Plcd4). Moreover, the gene kyphoscoliosis peptidase (Ky), which plays an important role in muscle growth, was also up-regulated in the skeletal muscle of the transgenic mice. The bar plots of the differentially expressed genes are shown in Figure S3, and all differentially expressed genes identified by RNA-seq are summarized in Table S1.

**Table 3:** Differentially expressed genes between myostatin propeptide transgenic and littermate control mice. (FPKM ≥ 5; fold change ≥ 2). inf represent the fold change cannot be calculated because the expression level was not detectable in wild-type mice.

Gene ID	Gene	Official Full Name	CN Value	TN Value	Fold Change	*p*-Value
**Genes Related to Cytoskeleton and Muscle Development**						
XLOC_010673	Myh6	Myosin heavy polypeptide 6	5.49	0.70	−7.83	0.00010
XLOC_000666	Mybph	Myosin binding protein H	14.77	57.60	3.90	0.00005
XLOC_003363	Rmst	Rhabdomyosarcoma 2 associated transcript	0.53	15.11	28.72	0.00005
XLOC_006460	Actg1	Actin gamma cytoplasmic 1	11.26	355.42	31.56	0.00005
XLOC_019664	Actc1	Actin alpha cardiac muscle 1	50.30	257.64	5.12	0.00005
XLOC_034484	Ky	Kyphoscoliosis peptidase (CD1)	3.26	8.81	2.70	0.00015
XLOC_026810	Actb	Actin beta	26.24	67.11	2.56	0.00020
XLOC_000205	Mstn-pro	Myostatin propeptide	32.03	409.35	12.78	0.00005
**Genes Related to Lipoprotein Metabolic Process and Intracellular Signaling Cascade**						
XLOC_034105	Apoa1	Apolipoprotein A-I	11.04	1.76	−6.27	0.00025
XLOC_019500	Dgkz	Diacylglycerol kinase zeta	22.14	90.10	4.07	0.00025
XLOC_000353	Plcd4	Phospholipase C delta 4	3.76	17.38	4.63	0.00010
XLOC_025320	Alb	Albumin	83.44	9.80	−8.51	0.00005
XLOC_002012	G0s2	G0/G1 switch gene 2	4.35	23.62	5.42	0.00005
**Genes for Immune Response**						
XLOC_007714	Ighm	Immunoglobulin heavy constant mu	27.24	73.44	2.70	0.00020
XLOC_007114	Tnfaip2	Tumor necrosis factor alpha-induced protein 2	3.90	17.93	4.60	0.00010
XLOC_031749	Trim12c	Tripartite motif-containing 12C	0.34	34.88	103.84	0.00025
**Genes for Oxidation Reduction**						
XLOC_006482	Cbr2	Carbonyl reductase 2	4.44	41.62	9.37	0.00010
XLOC_010336	Mss51	Mitochondrial translational activator	77.38	16.04	−4.83	0.00005
XLOC_020605	Kcnab1	Potassium voltage-gated channel shaker-related Subfamily beta member 1	2.86	11.06	3.87	0.00020
**Genes for Cell Proliferation**						
XLOC_001651	Ptprc	Protein tyrosine phosphatase receptor type C (CD45)	0.81	7.90	9.77	0.00005
XLOC_026557	Gm13841	Predicted gene 13841 (ribosomal protein L29)	0.00	8.32	inf	0.00005
**Genes of Proteolysis**						
XLOC_024383	Zmpste24	Zinc metallopeptidase STE24	5.67	27.67	4.88	0.00015
**Genes of Carbohydrate Phosphatase Activity**						
XLOC_018711	Pfkfb3	6-phosphofructo-2-kinase/fructose-2,6-biphosphatase 3	8.83	30.52	3.46	0.00005
**Genes of Integral to Membrane, Intrinsic to Membrane**						
XLOC_015439	Aqp4	Aquaporin 4	2.40	6.81	2.83	0.00020
**Genes of Extracellular Region**						
XLOC_025292	Csn1s2a	casein alpha s2-like A	0.00	8.54	inf	0.00005
**Others**						
XLOC_029580	Josd2	Josephin domain containing 2	109.35	22.64	−4.83	0.00005
XLOC_025924	Gm15459	Predicted gene 15459 (heat shock protein 8 pseudogene)	7.46	21.77	2.92	0.00005
XLOC_003234	Odf3l2	Outer dense fiber of sperm tails 3-like 2	14.39	57.11	3.97	0.00005

### 2.4. Function Annotation of the Differentially Expressed Genes

**Figure 3:**
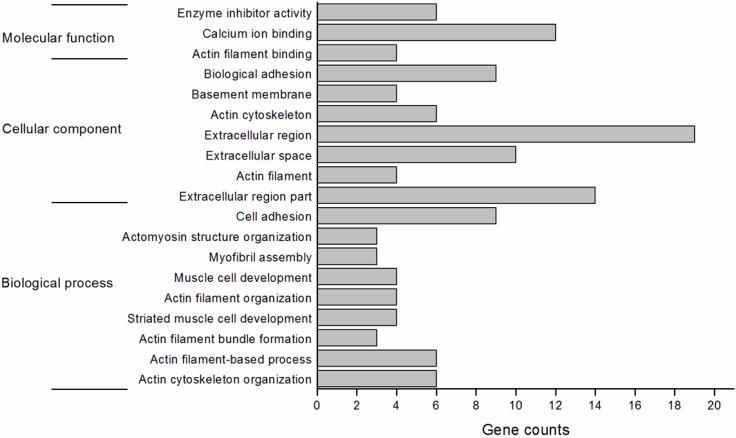
Overview of enriched gene ontology functional classifications of muscle differential expression genes between MSTN propeptide transgenic and wild-type mice.

To gain insight into the function of the differentially expressed genes in the hypertrophic skeletal muscle of the MSTN propeptide transgenic mice, the DAVID (Database for Annotation, Visualization and Integrated Discovery) website (http://david.abcc.ncifcrf.gov/) was used to identify the gene function with the following parameters: Count = 2 and EASE = 0.01. The gene ontology functional classification is shown in Figure 3. The functions of genes in biological processes, cellular components and molecular processes were annotated based on the GO categories. Most genes were enriched in two types of cellular components: the extracellular region and extracellular space. The genes enriched in biological processes were mainly involved in biological adhesion and cell adhesion. The genes enriched in molecular function were mainly associated with calcium ion binding.

### 2.5. Validation of the Differentially Expressed Genes by RT-qPCR

**Figure 4:**
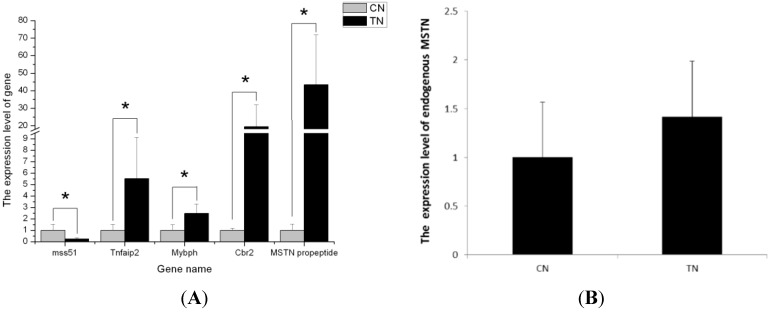
Q-PCR validation of genes from RNA-seq results between MSTN propeptide transgenic and control mice. All samples were normalized to 18S RNA. (**A**) Tnfaip2, Mybph, Cbr2 and MSTN propeptide were highly expressed in transgenic mice (TN) compared with control mice (CN). Mss51 had lower expression in transgenic mice (TN) compared with control mice (CN); (**B**) Endogenous myostatin (*C*-terminal mature peptide) mRNA levels in transgenic mice was slightly higher than the controls, but not significantly different from the control (*p* > 0.05). The error bars show the SD. Two-tailed *t*-test was used to calculate the significance of differentially expressed genes. Mark * in Figure 4A represent the expression level significantly different between two group (*p* < 0.05).

**Figure 5:**
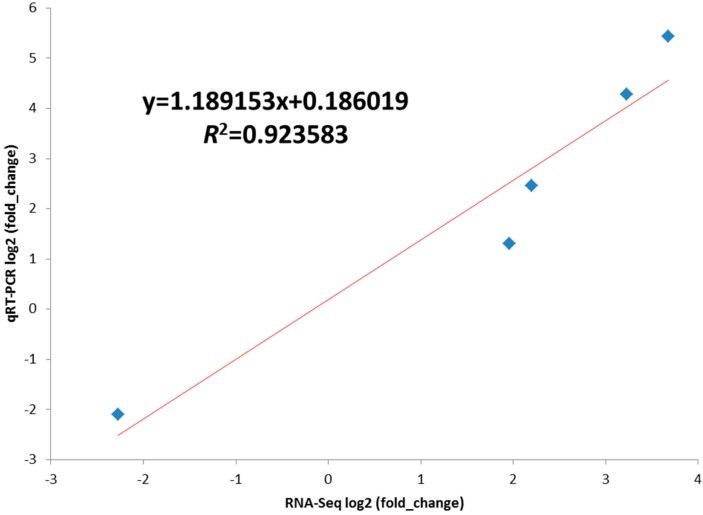
Line fit plot of Q-PCR results and RNA-Seq data for selected genes difference expression between MSTN propeptide transgenic and control mice. Linear regression model and *R*-Squared shown in the figure.

To verify the reliability of the sequencing data, five genes were randomly selected from Table 3 for RT-qPCR analyses (Figure 4). As expected, the gene expression patterns validated by RT-qPCR strongly correlated with the sequencing results (Pearson’s r correlation coefficient is 0.97591, Figure 5). Both RT-qPCR and solexa sequencing indicated that MSTN propeptide, Cbr2, Tnfaip2 and Mybph were up-regulated, while Mss51 was down-regulated in MSTN propeptide transgenic mice. Among the differentially expressed genes, the MSTN propeptide mRNA level increased by 12.78-fold according to solexa sequencing (Figure S4) and 43-fold according to the RT-qPCR analyses (Figure 4A). We also detected the expression level of endogenous MSTN; the endogenous myostatin expression did not significantly differ (*p* > 0.05) between the transgenic mice and their littermate wild-type controls (Figure 4B).

### 2.6. Signaling Pathway Analysis

**Figure 6:**
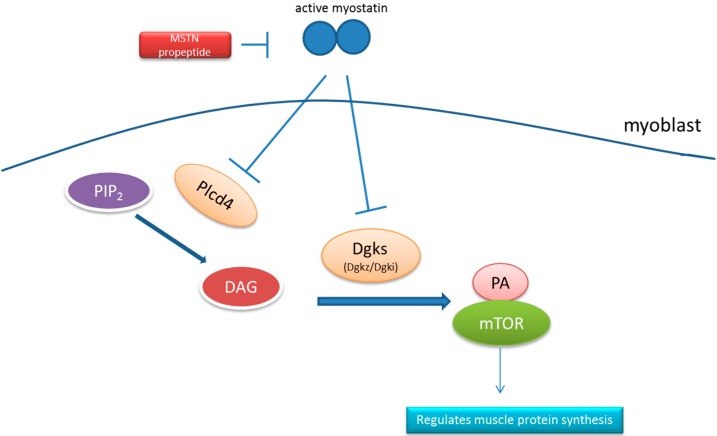
A theoretical model of the negative regulation of the PA signal pathway by myostatin in skeletal muscle. Plcd4 can catalyze hydrolyzes phosphatidylinositol 4,5-bisphosphate (PIP2) to inositol 1,4,5-trisphosphate (IP3) and DAG [32,33,34]. Diacylglycerol (DAG) can then be phosphorylated by Dgkz to produce phosphatidic acid (PA). Subsequently, PA binds to the FKBP12-rapamycine binding (FRB) domain of mTOR to induce muscle fiber hypertrophy [35,36].

To identify pathways that are involved in the regulation of muscle hypertrophy in the MSTN propeptide transgenic mice, we employed the DAVID website to analyze the differentially expressed genes with the following parameters: Count = 2 and EASE = 0.1. The pathways and related genes are listed in Table 4. This analysis identified the hypertrophic cardiomyopathy (HCM) and dilated cardiomyopathy signaling pathways, both of which regulate muscle growth and development. In addition, pathways associated with the maintenance of muscle structure and myocytes were identified, including the ECM-receptor interaction and focal adhesion pathways. Notably, the gene expression of three key enzymes, Dgki, Dgkz and Plcd4, of the phosphatidic acid (PA) signaling system was significantly enhanced in the skeletal muscle of the transgenic mice compared with their littermate controls (Figure 6). The hematopoietic cell lineage genes Itga4, cd3d and cd5 were also identified.

**Table 4:** The pathways regulated by differentially expressed genes.

Category	Term	Gene Counts	Genes
KEGG_PATHWAY	Hypertrophic cardiomyopathy (HCM)	5	Actb, Itga4, Actg1, Myh6, Acta
KEGG_PATHWAY	Dilated cardiomyopathy	5	Actb, Itga4, Actg1, Myh6, Acta
KEGG_PATHWAY	Focal adhesion	5	Lama1, Lamc3, Actb, Itga4, Actg1
KEGG_PATHWAY	Arrhythmogenic right ventricular cardiomyopathy (ARVC)	3	Actb, Itga4, Actg1
KEGG_PATHWAY	Phosphatidic acid signaling system	3	Dgki, Dgkz, Plcd4
KEGG_PATHWAY	Complement and coagulation cascades	3	C4bp, Proc, F12
KEGG_PATHWAY	ECM-receptor interaction	3	Lama1, Lamc3, Itga4
KEGG_PATHWAY	Hematopoietic cell lineage	3	Itga4, Cd3d, Cd5

## 3. Discussion

High-throughput sequencing technologies are currently emerging as a powerful tool to initially elaborate the genetic mechanisms responsible for a phenotype. Previous studies have detected genes related to the hypertrophic muscle observed in myostatin propeptide transgenic mice by employing microarray analysis [30]. The results identified 52 unique genes that are differentially expressed, and myostatin propeptide promotes adult muscle growth and maintenance by up-regulating the expression levels of myogenic regulatory factors and extracellular matrix components and down-regulating protein degradation-related genes as well as mitochondrial ATP synthesis-related genes [30].

To further illustrate the molecular mechanism and identify potential regulatory genes associated with the muscle hypertrophy caused by myostatin inhibition, we employed RNA sequencing technologies to study differences in the expression patterns between MSTN propeptide transgenic mice and wild-type littermate control mice at four months of age; these mice were younger than those used in the microarray study. In the current study, 132 differentially expressed genes were identified in MSTN propeptide transgenic mice compared with control mice based on the RNA sequencing technology and Cufflinks analysis method. Among these 132 genes, most were up-regulated in transgenic mice, and only 35 genes were down-regulated. Functional annotation showed that these differentially expressed genes mainly correlated with muscle development, lipid metabolism, energy metabolism and the immune system. Intriguingly, both of the abovementioned studies revealed that the function of some differentially expressed genes involves the extracellular region. Additionally, pathway analysis found four pathways in both studies: focal adhesion, ECM-receptor interaction, the regulation of the actin cytoskeleton, and cardiac muscle contraction. 

In the current study, we found that the expression levels of cytoskeletal and muscle growth and development-related genes were changed in the transgenic mice. Strikingly, Myh6 was down-regulated in the transgenic mice compared with the wild-type controls. A previous study showed that protein synthesis and muscle hypertrophy were enhanced when the expression of myh6 was repressed [37,38,39], suggesting that myh6 is a negative regulator of muscle mass. This finding is consistent with our results, which showed that Myh6 expression was decreased in muscle hypertrophy mice. Moreover, a previous study reported that the mitochondria number, the ratio of mitochondrial DNA to nuclear DNA, and the level of oxidative enzymes used to synthetize ATP all decreased in myostatin-deficient muscle [40]. Other studies showed that Myh6 had higher ATPase activity compared with other myosins [38,41]. Thus, the energy metabolism of myostatin propeptide transgenic mice may decrease due to a decrease in the expression of Myh6, which reduces ATP consumption. Unlike Myh6, the muscle growth-related genes Ky, Actb, Mybph, Actc1, RMST and Actg1 were up-regulated in our study. Among these genes, Ky (kyphoscoliosis peptidase) plays a vital role in muscle growth; the absence of Ky protein leads to muscular dystrophy [42,43]. A Ky mutation results in degenerative myopathy and loss of muscle hypertrophy responses [42]. Ky protein can interact with myosin-binding protein and filamin C [44]. Moreover, Myosin-binding protein H (Mybph) reportedly interacts with Rho kinase 2 (ROCK2) to affect the interaction of actin-myosin [45,46,47]. Additionally, Actb, Actc1 and Actg1 are three types of actins that participate in the maintenance of the cytoskeleton [48]. A functional analysis of these cytoskeletal and muscle development-related genes suggested that myostatin propeptide can enhance muscle mass via the Ky/Mybph/Actins pathway. 

Lipids play a critical role in signal transduction; they often act as a secondary messenger to activate downstream pathways [49]. We found that the overexpression of myostatin propeptide activated the phosphatidic acid (PA) signaling pathway, which generates signaling lipids and participates in the cell cycle and cell growth [50,51]. In the current study, we found that Plcd4 and Dgkz, two key enzymes of the PA signaling pathway, were up-regulated in myostatin propeptide transgenic mice. This result suggested a connection between the myostatin signaling and PA signaling pathways. Plcd4 is a member of the delta class of phospholipase C enzymes. Phospholipase C enzymes can catalyze hydrolyzing phosphatidylinositol 4,5-bisphosphate (PIP_2_) to two intracellular secondary messengers, inositol 1,4,5-trisphosphate (IP_3_) and diacylglycerol (DAG) [32]. Previous studies reported that DAG could be phosphorylated by Dgkz, and the phosphorylated DAG could be digested to phosphatidic acid (PA). PA can reportedly bind to mTOR to induce muscle fiber hypertrophy [52,53,54,55,56]. Other studies reported that myostatin mediates myoblast differentiation and myotube hypertrophy by inhibiting Akt/mTOR/p70S6 protein signaling [18,20,57]. Overall, we may infer that myostatin can negatively regulate the PA signing pathway to inhibit the mTOR signaling pathway (Figure 4). In addition to activating the mTOR signaling pathway, PA also activates p21-activated kinase 1 (PAK1) to initiate the release of RhoGDI from Rac1, and RhoGD1 subsequently changes the actin dynamics [58].

In biology, redox reactions can frequently store and release biological energy by electron transfer processes. In our study, we found that heme-binding protein Mss51, which is related to redox reactions, was down-regulated in myostatin propeptide transgenic mice. In yeast, Mss51 can regulate the biogenesis of cytochrome c oxidase (COX), the terminal electron transport chain (ETC) oxidase, thereby controlling energy production [59,60]. Previous studies showed that cytochrome c oxidase subunit VIc (Cox6c) expression is decreased in myostatin propeptide transgenic mice [30]. Although we did not detect differential Cox6c expression in our study, the expression of its upstream gene, Mss51, was decreased. Thus, we can infer that myostatin may influence ATP synthesis via Mss51. In addition, the gene Cbr2, which participates in neutralizing excessive reactive oxygen species (ROS), is also regulated by myostatin propeptide [61,62,63]. Myostatin induces the production of ROS, which decreases muscle mass by regulating antioxidant gene expression and mitochondrial biogenesis [64,65,66]. In combination with our results, these findings suggest that myostatin leads to muscle dystrophy by negatively regulating Cbr2 to increase ROS.

## 4. Experimental Section

### 4.1. Muscle Tissue Sample Collections

Myostatin propeptide cDNA under the control of rat myosin light chain 1 (MLC1) with its enhancer, SV40 poly A signal sequence, was constructed and served as the transgene construct, and transgenic mice were generated by standard microinjection techniques as previously described [28,29,30]. Male mice (hemizygous genotype for the transgene) from the high-expressing line were mated with B6SJL F1 wild-type females. The mice were housed in cages with a 12-h light/dark cycle, and the room temperature was maintained at 22 °C. The mice were weaned at four weeks of age and given free access to a standard diet (10% kcal fat, ME 3.85 kcal/g). All procedures and animal care were in accordance with the institution guidelines and approved by the Institutional Animal Care and Use Committee of the University of Hawaii, Honolulu, HI, USA. Male mice were sacrificed at four months of age after 8 h of fasting to sample and dissect the muscle tissue. Gastrocnemius muscle samples from both legs were immediately dissected from the carcass, quickly frozen in liquid nitrogen, and stored at −80 °C. Three transgenic male mice and two wild-type littermate male control samples were used for this study.

### 4.2. RNA Isolation and RNA Sequencing

Total RNA was extracted from freshly frozen muscle tissues using the TRIzol Reagent (Invitrogen, Carlsbad, CA, USA) according to the manufacturer’s instructions. A magnetic bead homogenizer was used to homogenize the tissue and TRIzol Reagent. The quality and concentration of RNA was detected by NanoDrop ND2000 (Thermo Fisher Scientific, Waltham, MA, USA) spectrophotometry and gel electrophoresis. Solexa sequencing was used to detect the differentially expressed genes in the gastrocnemius of the transgenic and control mice. Five cDNA libraries were constructed using the TruSeq Stranded Total RNA LT Sample Prep Kit (Illumina, Santiago, CA, USA). The library construction and solexa sequencing were performed by Genergy biological technology Limited company (Shanghai, China).

### 4.3. Quality Control for Raw Sequencing Data

The raw sequencing data were in a FASTQ file, and the data with clean reads were obtained by trimming the adapter contaminants and filtering the low-quality reads. A quality control tool, HTSeq (https://pypi.python.org/pypi/HTSeq), which can provide a quick impression of high throughput data, was utilized in our study. The quality control results are presented in the supplementary information.

### 4.4. Bioinformatics Analysis

The software Bowtie2 (http://bowtie-bio.sourceforge.net/index.shtml/) was used to merge the chromosome genomes of Mus musculus downloaded from the ENSEMBL database (ftp://ftp.ensembl.org/pub/release-75/fasta/mus_musculus/dna/) and convert them to the FM index, which can store massive genomic data and rapidly be searched. Tophat (http://ccb.jhu.edu/software/tophat/index.shtml), which uses Bowtie as an engine to align the sequencing reads to reference genome, was used to align the clean reads of each individual to the large genomes. Subsequently, the transcriptome was assembled with the Cufflinks software (http://cufflinks.cbcb.umd.edu/), and these assemblies were merged using Cuffmerge in the presence of the reference genome annotation (ftp://ftp.ensembl.org/pub/release-75/gtf/mus_musculus/). Cuffmerge provided a uniform basis to calculate the expression level of transcripts and genes at different conditions.

Cuffdiff, a separate program contained in Cufflink, was used to calculate the expression level of each individual and test the statistical significance between transgenic and control mice. Cuffdiff used FPKM (fragments per kilobase of transcript per million mapped fragments) method to calculate the expression level. In addition, CummeRbund was used to visualize and integrate all results provided by the Cuffdiff analysis. DAVID Bioinformatics Resources were used for gene annotation and pathway analysis.

### 4.5. Differential Expression Gene Validation by Real Time RT-PCR

Quantitative real-time PCR was performed to validate the differentially expressed genes identified by the Solexa sequencing data. Five differentially expressed genes were randomly selected for quantitative real-time PCR analysis. The Primer Premier 5.0 (PREMIER Biosoft International, Palo Alto, CA, USA) software was used to design the quantitative primers based on the cDNA sequence. RevertAid First Strand cDNA Synthesis Kit (Thermo Fisher Scientific, Waltham, MA, USA) was used for RNA reverse transcription following the manufacturer’s instructions, and DNase I and RNase-free (Thermo Fisher Scientific, Waltham, MA, USA) were used to treat the RNA sample to avoid genomic DNA contamination. The qPCR reaction was performed on a LightCycler 480 Real-Time PCR instrument (Roche, Penzberg, Germany) as follows: single cycle of denaturation at 95 °C for 5 min, 40 cycles of denaturation at 95 °C for 15 s, annealing at 60 °C for 15 s and extension at 72 °C for 15 s. SYBR Green (BIO-RAD, Hercules, CA USA) was used for the qPCR reaction, and all reactions were performed in triplicate. The 2^−ΔΔ*C*t^ method was used to calculate the relative expression levels between transgenic and control mice, and all expression levels were normalized to the expression of 18S RNA. Significant differences between groups were assessed with a two-tailed *t*-test. The difference between groups was considered significant when *p* < 0.05.

## 5. Conclusions

In this study, gene expression profiles associated with hypertrophic skeletal muscle in myostatin propeptide transgenic mice were investigated by RNA sequencing. A total of 132 genes were identified and used for function annotation and gene enrichment analysis. The novel up-regulated genes were related to hypertrophic cardiomyopathy, focal adhesion, and PA signaling pathway, and ROS removal processes, suggesting that myostatin directly affects these processes during the regulation of skeletal muscle growth and mass. Two new signaling pathways, the Ky/Mybph/Actins and PA signaling pathways, were uncovered, which may account for myostatin propeptide-induced muscle hypertrophy. Our results provide information to help further understand how myostatin inhibition causes skeletal muscle hypertrophy.

## Supplementary Materials

Supplementary materials can be found at http://www.mdpi.com/1422-0067/16/04/7976/s1.
