# Supplementary material for: RNA Sequencing Identifies Upregulated Kyphoscoliosis Peptidase and Phosphatidic Acid Signaling Pathways in Muscle Hypertrophy Generated by Transgenic Expression of Myostatin Propeptide
Source: Int J Mol Sci. 2015 Apr 9;16(4):7976–94. doi: 10.3390/ijms16047976 (PMC4425062; doi:10.3390/ijms16047976)
Supplement: Supplementary file 1 [file ijms-16-07976-s001.zip › ijms-73943-Supplementary Information/ijms-73943-supplementary.pdf]

Supplementary Information

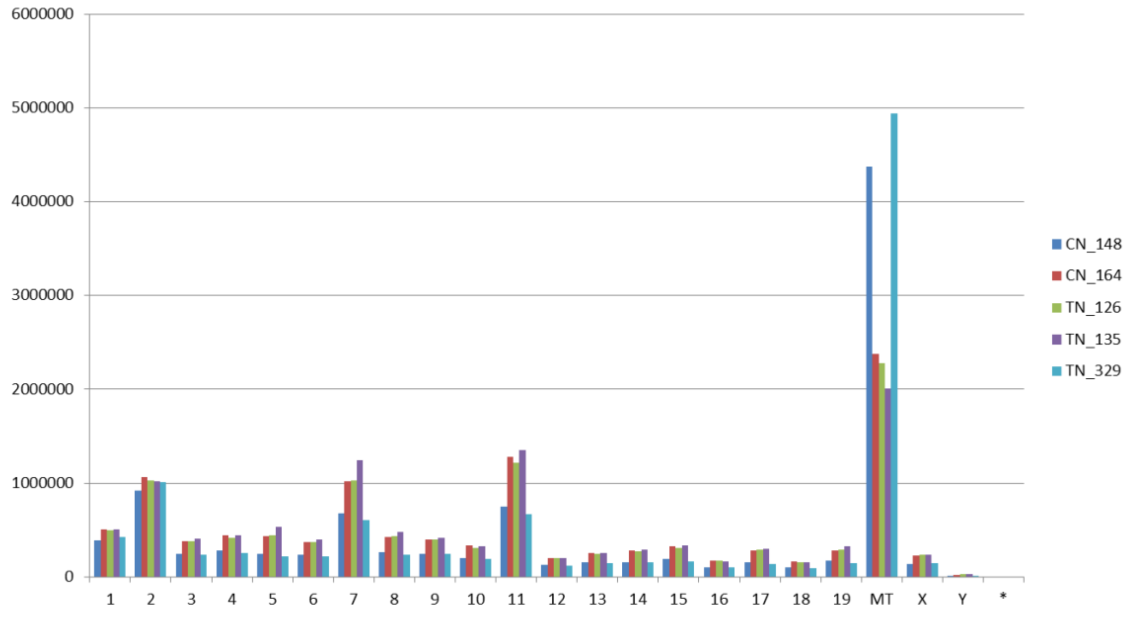

Figure S1. The abundance of mRNA reads mapped to each chromosome. The mark * represent non-chromosome, nonchromosomal FASTA file download from ENSEMBEL database contains DNA that has not been assigned a chromosome.

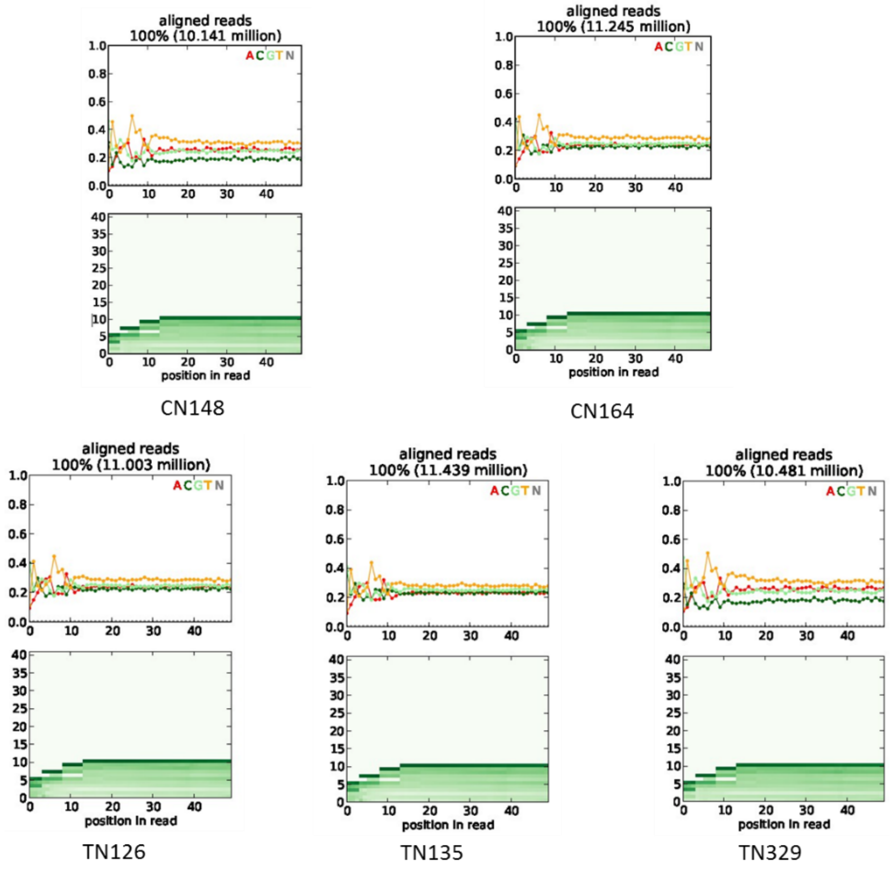

Figure S2. The sequencing quality assess of the five samples.

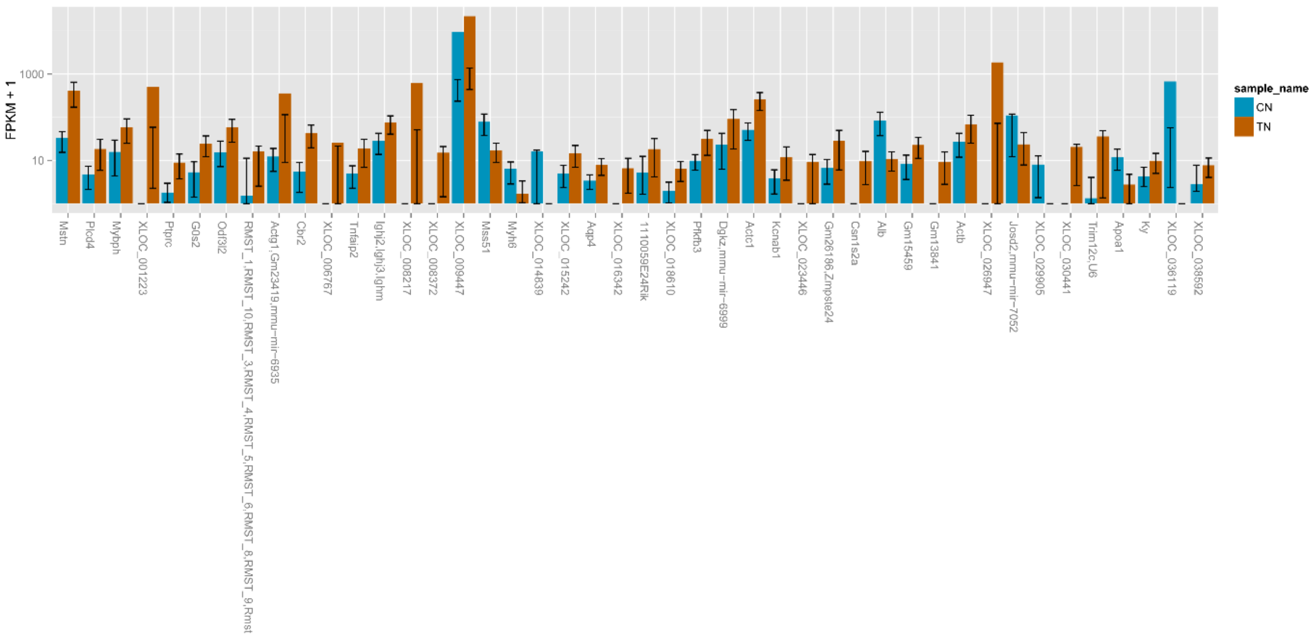

Figure S3. Bar plots for the significantly differentially expressed genes.

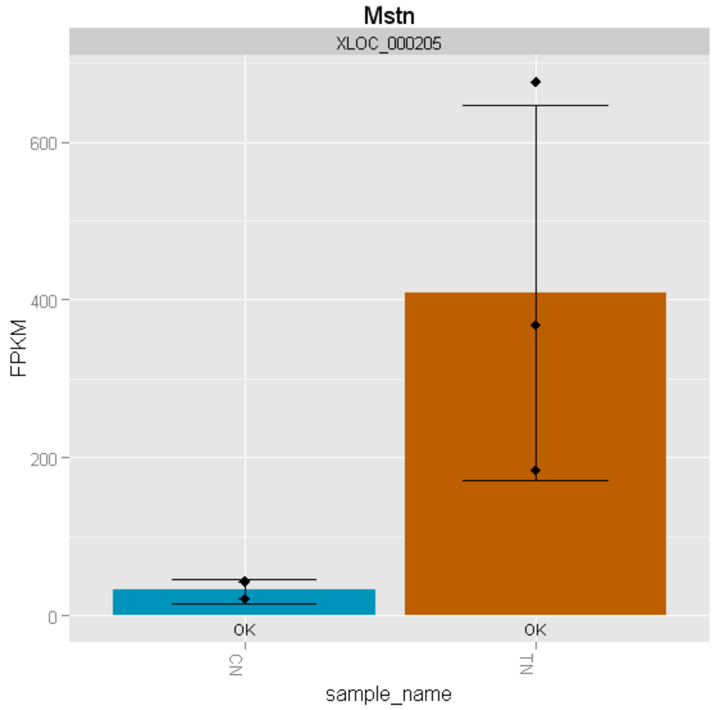

Figure S4. Bar plots of the expression level of MSTN propeptide.
